# The KIF1A homolog Unc-104 is important for spontaneous release, postsynaptic density maturation and perisynaptic scaffold organization

**DOI:** 10.1038/srep38172

**Published:** 2017-03-27

**Authors:** Yao V. Zhang, Shabab B. Hannan, Jeannine V. Kern, Doychin T. Stanchev, Baran Koç, Thomas R. Jahn, Tobias M. Rasse

**Affiliations:** 1 Junior Research Group Synaptic Plasticity, Hertie-Institute for Clinical Brain Research, University of Tübingen, Otfried-Müller-Str. 27, 72076 Tübingen 72076, Germany; 2 Graduate School of Cellular and Molecular Neuroscience, University of Tübingen, 72074 Tübingen, Germany; 3 The Picower Institute for Learning and Memory, Department of Biology and Department of Brain and Cognitive Sciences, Massachusetts Institute of Technology, Cambridge, MA 02139, USA; 4 CHS Research Group Proteostasis in Neurodegenerative Disease at CellNetworks Heidelberg University and DKFZ Deutsches Krebsforschungszentrum, Im Neuenheimer Feld 581, 69120 Heidelberg, Germany

## Abstract

The kinesin-3 family member *KIF1A* has been shown to be important for experience dependent neuroplasticity. In *Drosophila*, amorphic mutations in the *KIF1A* homolog *unc-104* disrupt the formation of mature boutons. Disease associated *KIF1A* mutations have been associated with motor and sensory dysfunctions as well as non-syndromic intellectual disability in humans. A hypomorphic mutation in the forkhead-associated domain of Unc-104, *unc-104*^*bris*^, impairs active zone maturation resulting in an increased fraction of post-synaptic glutamate receptor fields that lack the active zone scaffolding protein Bruchpilot. Here, we show that the *unc-104*^*bris*^mutation causes defects in synaptic transmission as manifested by reduced amplitude of both evoked and miniature excitatory junctional potentials. Structural defects observed in the postsynaptic compartment of mutant NMJs include reduced glutamate receptor field size, and altered glutamate receptor composition. In addition, we observed marked loss of postsynaptic scaffolding proteins and reduced complexity of the sub-synaptic reticulum, which could be rescued by pre- but not postsynaptic expression of *unc-104*. Our results highlight the importance of kinesin-3 based axonal transport in synaptic transmission and provide novel insights into the role of Unc-104 in synapse maturation.

Kinesins are microtubule based molecular motors that transport various cargos including membranous organelles, protein complexes and messenger RNAs^1^. Thus, they are of fundamental importance for the establishment, plasticity, injury response and survival of neuronal networks^2^,^3^. *C. elegans* Unc-104^4^,^5^, *Drosophila* Unc-104/Imac^6^ and the mammalian kinesin-3 family members KIF1A and KIF1B^7^,^8^ are the predominant motor proteins for the fast anterograde transport of membranous organelles. *KIF1A* knockout mice display severe neurological abnormalities including motor and sensory disturbances and die shortly after birth^8^. In *Drosophila* complete loss of *unc-104* function in motor neurons leads to an arrest of synaptogenesis and embryonic lethality^6^.

In humans recessive, autosomal dominant and spontaneous mutations in *KIF1A* have been associated with hereditary spastic paraplegia (HSP)^9^,^10^,^11^,^12^,^13^, and hereditary sensory and autonomic neuropathy type IIC (HSAN2C)^14^. Moreover, mutations in *KIF1B* has been implicated in Charcot-Marie-Tooth disease^15^. These diseases primarily affect nerve cells that have long axons and are thus most dependent on efficient cargo transport, consistent with kinesin-3’s important role for long-range intracellular trafficking. Apart from these symptoms in the peripheral nervous system, HSAN2C related mutations in *KIF1A* were also described to affect the central nervous system causing mental retardation and brain atrophy^9^,^16^,^17^. While *unc-104*’s mammalian homolog *KIF1A* is important in facilitating synaptogenesis in an experience dependent manner^18^, Unc-104 has been proposed to be part of the molecular machinery that regulates activity-dependent feedback in *Drosophila* photoreceptors^19^. Thus, defects in plasticity might be of major pathological relevance in the context of disease-related loss of *KIF1A* function.

We have previously reported a hypomorphic allele*, unc-104*^*bris*^ which causes partial loss of kinesin-3 function and thus permits detailed analysis of synapse maturation at the larval stage unlike the *unc-104* null mutant that dies at late-embryonic stage^6^. The recessive point mutation *unc-104*^*bris*^ at the forkhead associated (FHA) domain causes morphological changes at the neuromuscular junction (NMJ) such as increased NMJ length and synaptic bouton number as well as reduced bouton size^20^. In addition, *unc-104*^*bris*^have increased proportion of postsynaptic glutamate receptor fields that are unapposed by presynaptic active zone (AZ) scaffolding protein Bruchpilot (Brp)^20^,^21^,^22^,^23^. Furthermore, we have recently shown that these Brp-negative synapses lack all major AZ components investigated, such as Liprin-α, Cacophony and SRPK79D, suggesting a crucial role of *unc-104* in presynaptic maturation^23^.

Despite the mounting evidence on the consequences of *unc-104* loss-of-function on the maturation of the presynaptic terminal, little is known about its impact on postsynaptic development. In the present study, we examine functional and structural defects at *unc-104*^*bris*^ mutant NMJs with emphasis on structural deficits in the postsynaptic compartment. We found defects in synaptic transmission and altered glutamate receptor composition as a consequence of partial loss of kinesin-3 function. In addition, the postsynaptic compartments are characterized by the reduced abundance of discs large (Dlg), Dorsal B (DorB) and α-Spectrin (α-Spec) as well as reduced folding complexity of the subsynaptic reticulum (SSR). These defects can be rescued by the presynaptic but not postsynaptic expression of *unc-104*. Collectively, our data reveal new functions of kinesin-3 based transport in synapse development and may have important implications for our understanding of the molecular pathway underlying neurological diseases caused by impaired kinesin-3 function.

## Results

### Synaptic transmission is impaired in *unc-104* ^*bris*^ mutants

**Figure 1:**
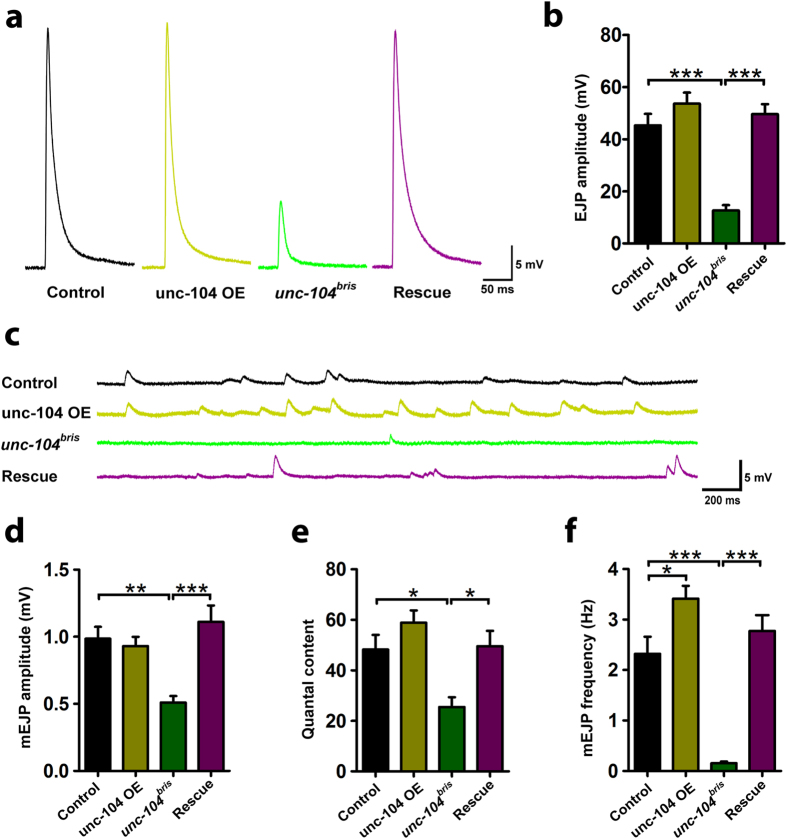
Impaired synaptic transmission in *unc-104*^*bris*^ mutant NMJs. (**a**) Representative traces of evoked release recorded from wild-type control, *unc-104*-OE, *unc-104*^*bris*^ and *unc-104*^*bris*^-Rescue group. (**b**) Quantification of average EJP amplitude. (**c**) Representative traces of spontaneous release recorded from wild-type control, *unc-104*-OE, *unc-104*^*bris*^ and *unc-104*^*bris*^-Rescue groups. (**d**–**f**) Quantification of mEJP amplitude (**d**) quantal content (**e**), and mEJP frequency (**f**) in the same groups. *Unc-104*^*bris*^ mutant NMJs showed significant reduction in all of the three quantified parameters, and these were rescued by pan-neuronal expression of *unc-104-mcherry* induced by the *elav-*Gal4 driver. 6–11 NMJs were quantified for each group. Martin’s correction for nonlinear summation (see material and methods) was applied for EJPs shown in (**b**) and for calculating quantal content. Statistical test: One-way ANOVA followed by Tukey’s Multiple Comparison Test. *P < 0.05; **P < 0.01; ***P < 0.001; n.s., P > 0.05. Error bars indicate the SEM. Genotypes: control (*w*^*1118*^), unc-104 OE (*elav-Gal4*/+;*UAS-unc-104-mcherry*/+;), *unc-104*^*bris*^ (;*unc-104*^*bris*^/*unc-104*^*d11024*^;), rescue (*elav-Gal4*/+;*unc-104*^*bris*^/*unc-104*^*d11024*^;*UAS-unc-104-mcherry*/+;).

*Unc-104*^*bris*^ mutant larvae demonstrate a distinct synapse maturation defect. At wild-type NMJs only the youngest synapses, in sum approximately 5%, lack the AZ organizing protein Brp^24^. In contrast, at the NMJs of *unc-104*^*bris*^ mutant larvae about 25% of the postsynaptic densities (PSDs) are unapposed by Brp^20^,^23^. In order to examine the functional consequences of impaired kinesin-3 function, we performed intracellular current clamp recordings at 3^rd^ instar larval NMJs. The amplitude of evoked and miniature excitatory junctional potentials (EJPs and mEJPs) was reduced by 72% and 50% respectively in *unc-104*^*bris*^ mutants compared to controls (Fig. 1a–d). The quantal content, which is an estimation of the number of synaptic vesicles (SVs) released per evoked release event, was decreased by 47% at *unc-104*^*bris*^ mutant NMJs (Fig. 1e). Furthermore, we observed a 20-fold reduction in mEJP frequency in *unc-104*^*bris*^ larvae. All above described functional defects were rescued by ectopic pan-neuronal expression of UAS-*unc-104-mcherry* (Fig. 1c,f). Expression of the rescue construct in the control background did not affect junctional potentials but resulted in a 42% increase in mEJP frequency (Fig. 1c,f).

### *Unc-104* ^*bris*^ mutants display altered glutamate receptor composition

**Figure 2:**
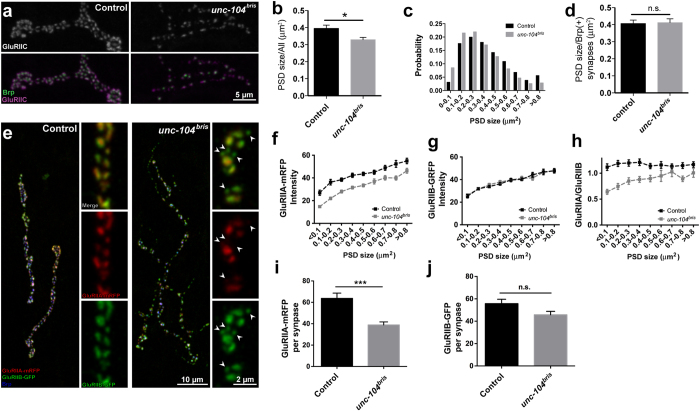
Impaired glutamate receptor composition at *unc-104*^*bris*^ mutant NMJs. (**a**) Representative confocal images of control and *unc-104*^*bris*^ mutant NMJs marked by the presynaptic AZ protein Brp (green) and postsynaptic GluR (grey & magenta). (**b**) Average PSD size of *unc-104*^*bris*^ mutant NMJs is reduced compared to the control (control: 0.395 ± 0.019 μm^2^; *unc-104*^*bris*^: 0.329 ± 0.013 μm^2^. P < 0.05). (**c**) PSD size distribution of control and *unc-104*^*bris*^ mutant NMJs. There is an increased proportion of small (<0.3 μm^2^) and decrease of larger (>0.3 μm^2^), likely mature synapses in *unc-104*^*bris*^ mutants. (**d**) The sizes of PSDs apposed by presynaptic Brp are unchanged between control and *unc-104*^*bris*^ mutants (control: 0.407 ± 0.020 μm^2^; *unc-104*^*bris*^: 0.412 ± 0.022 μm^2^. P > 0.05). (**e**) Confocal images of neuromuscular synapses stained with Brp (blue) in control and *unc-104*^*bris*^ mutant larvae expressing GluRIIA-mRFP (red) and GluRIIB-GFP (green). GluRIIA was reduced in *unc-104*^*bris*^ mutant synapses (arrowheads). (**f–h**) Quantification of GluRIIA-mRFP intensity, GluRIIB-GFP intensity and GluRIIA/GluRIIB ratio at PSDs in control and *unc-104*^*bris*^ mutant NMJs, grouped by synapse size. (**f**) GluRIIA-mRFP intensity in *unc-104*^*bris*^ was significantly lower than control in all size groups. (**g**) In contrast, GluRIIB intensity in control and *unc-104*^*bris*^ mutants PSDs were comparable. (**h**) As a result, *unc-104*^*bris*^ mutant PSDs showed lower GluRIIA/GluRIIB ratio in all size groups, with small synapses showing stronger impairment. (**i**) Average size of GluRIIA-mRFP fields. (**j**) Average size of GluRIIB-GFP fields. For **b**–**d** and **f**–**h**, Number of NMJs analyzed: N ≥ 9 for all size groups. Statistical test: Mann-Whitney test. *P < 0.05; n.s., P > 0.05. Error bars indicate the SEM. Genotypes: control (;*glurIIA-mrfp, glurIIB-gfp*/+;), *unc-104*^*bris*^ (;*unc-104*^*bris*^/*unc-104*^*d11024*^;*glurIIA-mrfp, glurIIB-gfp*/+;).

Multiple presynaptic structural defects have so far been identified in the context of loss of *unc-104* function^6^,^20^,^23^. However, the importance of Unc-104 for postsynaptic development is largely unexplored. We next investigated whether impaired synaptic transmission at *unc-104*^*bris*^ mutant NMJs might be caused by altered postsynaptic glutamate receptor composition and/or clustering. To this aim we first performed immunohistochemistry at the NMJ using antibodies against Brp and obligate glutamate receptor subunit IIC (GluRIIC). We observed a 17% reduction of the average GluR field size at the NMJ (Fig. 2a,b). The reduction in size is reflected by plotting all GluR fields based on their size, where *unc-104*^*bris*^ mutant NMJs have an increased percentage of small GluR fields (Fig. 2c). To address the role of presynaptic Brp presence on the size of GluR fields, we limited our size analysis only to those GluR fields that were positive for Brp. Intriguingly, we did not observe a difference in GluR field size in the subset of synapses that were positive for Brp (Fig. 2a,d).

Two types of ionotropic glutamate receptors have been identified at the *Drosophila* NMJ synapses. Apart from the three obligatory subunits, they either contain GluRIIA or GluRIIB as a fourth subunit to form a functional heterotetrameric receptor^25^. The *Drosophila* NMJ contains a mixture of IIA- and IIB-type glutamate receptors. We sought to assess whether differential incorporation and stabilization of glutamate receptor complexes could be observed at *unc-104*^*bris*^ mutant NMJs. To visualize GluRIIB and GluRIIA, we utilized a double transgenic line expressing GluRIIB-GFP and GluRIIA-mRFP under the control of their native promotors^24^,^26^. The intensity of GluRIIA-mRFP was reduced in *unc-104*^*bris*^ mutant synapses (Fig. 2e, arrowheads and f). Localization and abundance of GluRIIB-GFP remained unchanged in *unc-104*^*bris*^ mutants (Fig. 2e, arrowheads and g). It has been reported that receptor composition changes during synapse maturation at *Drosophila* NMJ: IIA-type receptors are predominant in immature synapses, whereas the IIA- and IIB-type receptor ratios are balanced as synapses mature^26^. We quantified the IIA- and IIB-type receptors for each PSD and found that consistent with previous reports, small synapses in control group were “IIA-rich” compared with mature ones which had a more balanced glutamate receptor composition (Fig. 2h). In contrast, IIA-receptors are not enriched at small synapses of *unc-104*^*bris*^ mutant larvae (Fig. 2h). Irrespective of their size, synapses of *unc-104*^*bris*^ mutant larvae contain less IIA-type receptors than control (Fig. 2i), while no changes were observed in the amount of GluRIIB per synapse between control and *unc-104*^*bris*^ mutants (Fig. 2j). IIA- and IIB-type glutamate receptors exhibit distinct electrophysiological properties^25^,^27^,^28^,^29^, with the IIA-type receptors showing higher single channel conductance than IIB-type receptors^28^. Therefore, the altered receptor composition may contribute to the decreased EJP and mEJPs sizes in *unc-104*^*bris*^ mutants.

### Impaired abundance of post synaptic markers in *unc-104* ^*bris*^ mutants

**Figure 3:**
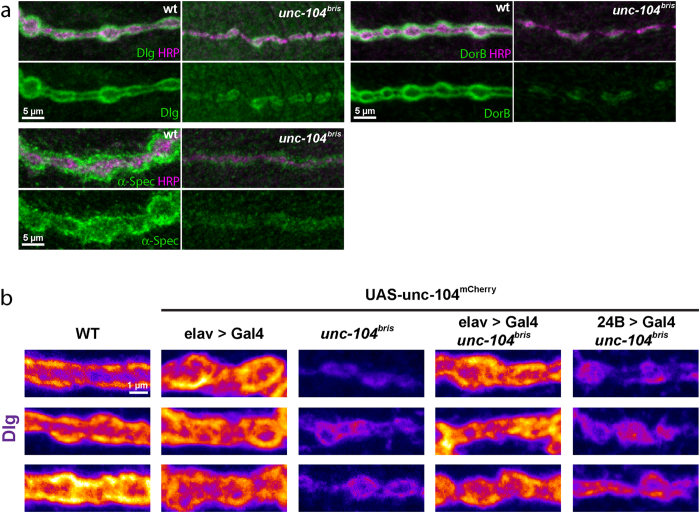
Reduced intensity of postsynaptic markers in *unc-104*^*bris*^ mutants. (**a**) A stretch of boutons stained with anti-HRP antibody (green) and antibodies against disc large (Dlg, magenta), Dorsal B (DorB, magenta) and α-Spectrin (α-Spec, magenta) showing reduced intensities at the subsynaptic reticulum. (**b**) Reduced Dlg expression in *unc-104*^*bris*^ mutant larvae. Confocal excerpts of NMJ 4 in A2 segment immuno-stained with antiserum to the scaffold protein Dlg. All genotypes were taken with the same laser intensity. The reduced expression of Dlg in *unc-104*^*bris*^ mutant larvae can be rescued by the pan-neuronal expression of *unc-104-mcherry*. Postsynaptic expression of *unc-104-mcherry* induced by *24B*-Gal4 results in no change in the expression of Dlg compared to *unc-104*^*bris*^ mutants.

Membrane associated guanylate kinase-like proteins (MAGUKs) including PSD protein 93 and 95 (PSD-93, PSD-95), SAP97 and SAP102 are thought to be key organizers of molecular scaffolds at excitatory synapses^30^. We examined the localization of Dlg^31^, the *Drosophila* homolog of PSD-95, at *unc-104*^*bris*^ mutant synapses to further investigate the impact of reduced Unc-104 function on postsynaptic development. We found that Dlg was severely reduced at *unc-104*^*bris*^ mutant NMJs (Fig. 3a). Next, we examined two additional postsynaptic markers, the *Drosophila* NF-κB protein DorB^31^,^32^ and the SSR enriched, cytoskeletal protein α-Spec^33^. Similar to Dlg, the levels of both DorB and α-Spec were also reduced at the SSR (Fig. 3b,c).

Reduced levels of synaptic markers observed in *unc-104*^*bris*^ could be a result of loss of Unc-104 in the presynaptic or postsynaptic compartment or both. Thus, we ectopically expressed UAS-*unc-104-mcherry* in the *unc-104*^*bris*^ background either pan-neuronally using *elav-*Gal4 or in muscles using *24B*-Gal4. Presynaptic but not postsynaptic expression of Unc-104 was able to restore normal Dlg abundance in mutant NMJs (Fig. 3b). Our results suggest that impaired pre-, and not postsynaptic, Unc-104 function is upstream of the partial loss of postsynaptic markers in *unc-104*^*bris*^ mutants.

### *Unc-104* ^*bris*^ mutants display gross morphological changes in SSR membranes

**Figure 4:**
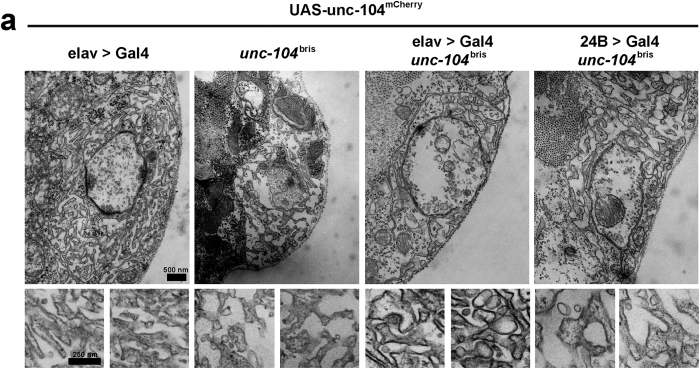
Subsynaptic reticulum development defects in *unc-104*^*bris*^ mutants. (**a**) Electron micrographs of NMJ 4 in A2 segment. In the control group the presynaptic “T-bar” structures are clearly visible and the subsynaptic reticulum (SSR) has a compact shape. The boutons of *unc-104*^*bris*^ larva are considerably smaller and the SSR is less compact. Pan-neuronal expression of *unc-104-mcherry* rescues this defect. Postsynaptic expression of *unc-104-mcherry* with *24B*-Gal4 does not improve the phenotype.

Type I boutons are surrounded by the SSR, which consists of densely stacked muscle membranes that are thought to be important for the stability of the neuromuscular terminal^31^,^34^. Loosening and vacuolization of the SSR has been described in NMJ dismantling during metamorphosis^35^. Given that partial loss of Dlg has been associated with reduced convolution of SSR membranes^31^,^36^, we investigated the ultrastructure of the extrasynaptic compartment of larval NMJs using electron microscopy. While wild-type boutons were surrounded by a tight array of muscle membranes, the SSR structure was less dense in *unc-104*^*bris*^ mutant NMJs suggesting that partial loss of Unc-104 function results in gross morphological changes in postsynaptic compartments (Fig. 4a). The ultrastructure of the SSR could be rescued by pre- but not by postsynaptic expression of *unc-104* cDNA, suggesting that SSR defects are secondary to loss of presynaptic Unc-104 function (Fig. 4a). Taken together, our results show that partial loss of Unc-104 function resulted in reduced levels of postsynaptic proteins and impaired SSR morphology.

## Discussion

*Unc-104*^*bris*^ mutant NMJs are characterized by a strong reduction in EJP amplitude (Fig. 1a,b). This is consistent with previous reports that *unc-104*^*bris*^ mutant larvae are severely impaired in locomotion; larval lethality is preceded by almost complete paralysis at L3 stage^20^. Notably, the strong reduction in EJP amplitude (Fig. 1a,b) and the number of vesicles released (Fig. 1d) per action potential are not being partially compensated in the postsynaptic compartment by concomitant increase in the mEJP amplitude (Fig. 1c), i.e. the response per vesicle. This is surprising as it has been previously reported that upon decrease in quantal content due to lack of Brp, mEJP amplitudes are scaled up^37^. Loss of Brp from a subset of synapses is a primary defect in *unc-104*^*bris*^ larvae^20^.

We observed a strong reduction of mEJP frequency at *unc-104*^*bris*^ mutant NMJs (Fig. 1c,f). Kinesin-3 is the major transporter of SVs, and previous reports show several SV markers are severely reduced at *unc-104* mutant NMJs^6^,^23^. Loss of SVs from the NMJ as well as alterations in the size of the readily releasable pool are possible causes for the decreased mEJP frequency at *unc-104*^*bris*^ mutant NMJs. While there is direct ultrastructural evidence for the loss of SVs from boutons in *unc-104* null mutant embryos^6^, we did not observe any reduction in the amount of SVs at central synapses in *unc-104*^*bris*^ mutant larvae^23^. Interestingly, while presynaptic over-expression of the UAS-*unc-104-mcherry* transgene in wild-type background did not cause any changes in EJP or mEJP amplitudes, it resulted in an increase in mEJP frequency (Fig. 1c,f). These data suggest that the frequency of spontaneous release is likely particularly sensitive to the abundance of readily releasable pool of SVs at the NMJ which might be regulated by kinesin-3 based axonal transport.

In the nervous system, homeostatic signaling is an essential feedback mechanism to ensure stable synaptic activity in a highly variable environment. Homeostatic signaling has been shown to compensate for perturbation of synaptic excitability through multiple mechanisms including changing the efficacy of SV release, ion channel density and neurotransmitter receptor composition^38^,^39^,^40^,^41^. The conductivity of IIA-type glutamate receptors is much larger than IIB-type receptors, and change in the ratio between these two receptors has been shown to modulate quantal size^27^. Thus, a change in receptor composition is a potential mediator of postsynaptic homeostasis. Synaptic transmission blockage via presynaptic expression of TNT leads for example to higher abundance of the GluRIIA at the PSD^26^. Likewise, the ratio of GluRIIA/GluRIIB containing glutamate receptors changes in *dsyd-1* mutants due to a simultaneous increase in GluRIIA and decrease in GluRIIB abundance^42^. However, no such change was observed in *liprin-α* mutants, that display a similar reduction in glutamate release^42^. Alternatively, this phenotype might thus be caused by a loss of a maturation signal that is present in *liprin-α* mutants, but lacking in *dsyd-1* mutants. Despite the severely impaired presynaptic AZ assembly^23^, no sign of postsynaptic compensation is observed in *unc-104*^*bris*^ mutants. Hence, the observed reduction in GluRIIA clustering is likely responsible for the decreased mEJP amplitude, and in turn contributes to the impaired EJP (Fig. 1a–c).

We have previously shown that the *unc-104*^*bris*^ mutation causes alteration of the gross NMJ structure, resulting in impaired synapse maturation along with severe reduced anterograde transport of SVs and dense core vesicles^20^,^23^. Interestingly, expression of Rab3, an SV-associated protein that is depleted from the nerve terminals of *unc-104*^*bris*^mutants partially ameliorates presynaptic and postsynaptic defects^23^. This suggests that some of the phenotypes observed in *unc-104*^*bris*^ mutants are likely due to the depletion of Unc-104 cargo at presynaptic terminal. The present study shows a severe loss of mEJP frequency in *unc-104*^*bris*^ NMJs (Fig. 1c,f). Given that mEJPs are an instructive signal that guides synaptic development^43^, some of the observed morphological defects in *unc-104*^*bris*^could be a result of reduced mEJP frequency (Fig. 1c,f). As presynaptic expression of Unc-104 rescues postsynaptic Dlg and SSR phenotypes (Figs 3b and 4a), it would be interestingly to address in a future study, if this rescue is dependent on specific presynaptic Unc-104 cargo.

Dlg, one of the major proteins localized to the SSR, is a known interaction partner of Gtaxin^44^, the *Drosophila* homolog of mammalian syntaxin-18, which regulates endoplasmic reticulum (ER) membrane trafficking^45^. It is intriguing to postulate that that Dlg and Gtaxin may cooperatively regulate addition of ER membrane to the SSR^46^, and via this membrane trafficking pathway ER characteristics might be transferred to the SSR^46^. Consistently, ample evidence suggests that local translation might occur at the SSR^47^,^48^,^49^. This raises the exciting possibility that the reduced SSR complexity observed at *unc-104*^*bris*^ mutant NMJs (Fig. 4) might be a result of impaired membrane addition to the SSR. The insufficiently developed SSR at *unc-104*^*bris*^ mutant NMJs might be less efficient in local translation of key proteins required for synaptic development. Consistent with this notion, the glutamate receptor subunit IIA, which is strongly reduced at *unc-104*^*bris*^ mutant NMJs (Fig. 2f), has been shown to be translated in local, subsynaptic translation aggregates^47^.

The membrane of insect muscles is invaginated at most synapses to frame a complex array of tubes and folds, the SSR. Although it is morphologically similar to subsynaptic folds that have been proposed to be important to amplify synaptic signals at the vertebrate NMJ, its primary cellular function might be different^50^. At the *Drosophila* NMJ, the SSR gradually develops at Type I boutons. It is more pronounced at Type Ib boutons, displaying more fully developed and deeper stacked SSR than Type Is terminals^34^,^51^. Type II and III fibers that often run parallel with Type I innervations might be partially embedded in the SSR emerging from the Type I innervation but do not form a multi-layered complex SSR on their own^51^. The SSR is also absent from synapses at indirect flight muscles^50^, suggesting that its role is modulatory rather than essential for neurotransmission. We found that the SSR in *unc-104*^bris^ mutant larvae is less dense than in control larvae (Fig. 4). While loosening of the SSR has been associated with NMJs dismantling during metamorphosis^35^, it is not very likely that the reduced SSR complexity at *unc-104*^bris^ mutant NMJs is due to ongoing neurodegeneration^20^.

Similar with a previously described HSP Type 10 (SPG10) *Drosophila* model, larval locomotion was also severely impaired in *unc-104*^bris^ mutant larvae^20^,^52^. However, while robust structural defects were observed upon disturbance of Kinesin-1 based transport^52^, no signs of synapse dismantling were observed in *unc-104*^bris^ mutant larvae^20^. Therefore it is more likely that the observed reduction of SSR associated proteins (Fig. 3a–c) and the reduction of SSR density (Fig. 4) at *unc-104*^bris^ mutant NMJs reflects a defect in synapse maturation and/or plasticity rather than being a sign of neurodegeneration.

The degree of behavioral impairment and the lethality of *unc-104*^*bris*^ larvae is similar to those described for a *Drosophila* model for SPG10 caused by dominant negative mutations in kinesin heavy chain (*khc*), the *Drosophila* homolog of human *KIF5A*^52^. Notably, although both disease models are characterized by a partial loss of kinesin function related to two forms of HSPs, different pathological progressions were observed. SPG10 model larvae display a pronounced dystonic posterior paralysis that is reminiscent of the ascending paralysis observed in SPG10 patients^52^. While SPG30 is also an ascending motoneuron disease that firstly and primarily affects nerve cells that innervate the feet of the patients, *unc-104*^*bris*^ larvae suffer from a more generalized paralysis, i.e. NMJs in segments innervated by longer axons seem not to be more severely affected^20^. This complex phenotype highlights that more detailed studies will be necessary to fully dissect the specific impact of kinesin-3 dysfunction on neural function and human pathology.

While truncation of *KIF1A* has been associated with another neurological disease HSAN2C^14^, various mutations of *KIF1A* have been associated with SPG30 as well as non-syndromic intellectual disability accompanied by variable additional symptoms including progressive encephalopathy and brain atrophy^9^,^16^,^17^,^53^,^54^. Recently, an amino acid exchange (S69L), proposed to be important for the ATP binding of KIF1A, has been shown to underlie SPG30^10^. A detailed comparison of different disease related mutations may give valuable insight on the specific impact of disrupting the function of different domains of KIF1A.

The amino acid mutated in *unc-104*^*bris*^ larvae has been suggested to further stabilize kinesin-3 dimers by electrostatic interaction with the E499 residue^55^. Defects observed in *unc-104*^*bris*^ larvae are not restricted to the axonal compartment, but include defects in dendrite maturation in sensory neurons^20^, a phenotype most relevant in the context of HSAN2C. We thus suggest that the animal disease model presented herein, rather than being a precise model of any of the human diseases associated with KIF1A dysfunction, might have broad implication for neurological diseases that are associated with impaired stability of kinesin-3 dimer including of HSP, HSAN2C and intellectual disability.

## Methods

### Fly Stocks

Flies were cultured on standard soft media seeded with live yeast at 25 °C unless otherwise indicated. *w*^*1118*^, *elav-*Gal4, *24B*-Gal4 and *unc-104*^*d11024*^ were obtained from the Bloomington *Drosophila* Stock Center. GluRIIA-mRFP^24^ and GluRIIB-GFP^26^ with endogenous promoters were obtained from Stephan Sigrist (FU Berlin). UAS-*unc-104-mcherry* was a generous gift from Thomas Schwarz (Harvard University).

### Immunohistochemistry and microscopy

Middle 3^rd^ instar stage larvae were dissected in Ca^2+^-free HL3 solution and fixed in 4% formaldehyde in PBS for 3 minutes (for staining with native fluorescent proteins) or for 10 minutes (for staining with only immunofluorescent labeling). Correct NMJs were identified as previously described^56^. Primary antibody incubation was done overnight at 4 °C in PBS containing 0.05% Triton-X and 5% normal goat serum. Fillets were then washed and incubated with fluorescent-conjugated secondary antibodies at room temperature for 2 hours. Larval fillets were mounted on a glass slide in mounting medium (Vectashield, Vector). Primary antibodies used were: mouse monoclonal anti-Dlg at 1:100, mouse monoclonal anti-Brp (NC82) at 1:100, mouse anti-α-Spec (3A9) at 1:100 (Developmental Studies Hybidoma Bank), rabbit anti-GluRIIC at 1:2000 (Stephan Sigrist), rabbit anti-DorB at 1:4000 (Steven Wasserman). Fluorescent-conjugated secondary antibodies used were: goat anti-mouse Alexa 488 or Alexa 568 and goat anti-rabbit Alexa 488 or Alexa 568 (Molecular Probes) or goat anti-mouse Atto 647 (Sigma). Goat anti-HRP conjugated with Cy3 (Dianova) was added together with secondary antibodies. All fluorescent-conjugated antibodies were diluted at 1:500. Images were captured and processed essentially as previously described^20^,^23^,^57^,^58^. In detail, a Zeiss LSM 710 confocal microscope with a 40x plan apochromat 1.3 N.A. oil objective and the ZEN software was used to acquire images at voxel size: 100 nm * 100 nm * 500 nm; pinhole: 1 AU, average: 2–4. ImageJ 1.43 (NIH) was used for image processing and analysis.

### Electron Microscopy

Sample preparation and image acquisition were performed essentially as previously described^52^. In brief, fillets were fixed with 4% PFA (in PBS) for 10 min at room temperature followed by fixation in 2.5% glutaraldehyde (in PBS) overnight at 4 °C. Next, samples were treated with 1% osmium tetroxide in 100 mM phosphate buffer, pH 7.2, for 1 h on ice. Larval fillets were rinsed with water, treated with 1% aqueous uranyl acetate (UA) for 1 h at 4 °C, dehydrated through a graded series of ethanol concentrations, and stored in liquid Epon overnight. Next, muscles 4 of segment 4 were dissected with sharp insect pins, embedded in Epon, and polymerized for 48 h at 60 °C. Ultrathin sections were stained with UA and lead citrate and viewed in a Philips CM10 electron microscope.

### Electrophysiology

Current clamp intracellular recordings were performed on muscle 6 segment A2 of mid 3^rd^ instar stage larvae as previously described^52^. The larvae were pinned and stretched in a Sylgard-coated perfusion chamber and visualized on an Olympus BX51WI microscope. “Bee-stinger” sharp electrodes (10–15 MΩ), made of borosilicate glass (outer diameter 1.5 μm) were filled with 3 M KCl. Only cells with resting potentials between −55 and −80 mV and input resistance higher than 4 MΩ were included in the analysis. Recordings were performed in HL3 Stewart saline^59^ containing (in mM): 70 NaCl, 5 KCl, 20 MgCl_2_, 10 NaHCO_3_, 5 trehalose, 115 sucrose, and 5 HEPES; the concentration of Ca^2+^ was 1, pH adjusted to 7.2. All experiments were performed at 18 °C. Stimulation of the segmental nerve was executed by pulling the cut end of the nerve into a self-made suction electrode (5–6 μm in diameter) filled with HL3 and passing a brief (0.3 ms) bi-polarizing pulse across the nerve. Stimulation was accomplished with an ISO-STIM 01D stimulus isolation unit (NPI electronics GmbH, Tamm, Germany). The signal was acquired with an Axoclamp 900A amplifier (Axon Instruments), digitized with a Digidata 1440A analog to digital board, and recorded with a PC using pClamp 10. 3 (Axon Instruments), and analyzed with AxoGraph X software. The amplitudes of the EJPs were corrected for nonlinear summation. The quantal content was estimated by dividing the averaged corrected EJP by the averaged mEJP amplitude^60^.

### Statistical Analysis

Statistical analysis was performed using the software PRISM 6. Sample errors are given as standard error of the mean (SEM). The following alpha levels were used for all tests: *p < 0.05; **p < 0.01; ***p < 0.001. Data were first tested for normality and then analyzed by either the student’s *t*-test for two groups or by a one-way analysis of variance followed by a Tukey’s multiple comparison test. Non-normally distributed data were analyzed by using either a Mann-Whitney test for two groups or a Kruskal-Wallis test for multiple groups.

### Use of experimental animals and human subjects

This study did not involve the use of human subjects or samples from human donors. All experiments were performed in accordance with University guidelines and regulations.

## Additional Information

**How to cite this article:** Zhang, Y. V. *et al*. The KIF1A homolog Unc-104 is important for spontaneous release, postsynaptic density maturation and perisynaptic scaffold organization. *Sci. Rep.* **7**, 38172; doi: 10.1038/srep38172 (2017).

**Publisher's note:** Springer Nature remains neutral with regard to jurisdictional claims in published maps and institutional affiliations.
